# Proteomic Analyses of Whitefly-Begomovirus Interactions Reveal the Inhibitory Role of Tumorous Imaginal Discs in Viral Retention

**DOI:** 10.3389/fimmu.2020.01596

**Published:** 2020-08-04

**Authors:** Jing Zhao, Tao Guo, Teng Lei, Jia-Chen Zhu, Fang Wang, Xiao-Wei Wang, Shu-Sheng Liu

**Affiliations:** Ministry of Agriculture Key Laboratory of Molecular Biology of Crop Pathogens and Insects, Institute of Insect Sciences, Zhejiang University, Hangzhou, China

**Keywords:** whitefly, TYLCV, interaction, Tid, antiviral infection

## Abstract

In nature, plant viruses are mostly transmitted by hemipteran insects, such as aphids, leafhoppers, and whiteflies. However, the molecular mechanisms underlying the interactions between virus and insect vector are poorly known. Here, we investigate the proteomic interactions between tomato yellow leaf curl virus (TYLCV, genus *Begomovirus*, family *Geminiviridae*), a plant virus, and its vector whitefly (*Bemisia tabaci*) species complex. First, using a yeast two-hybrid system, we identified 15 candidate whitefly proteins interacting with the coat protein of TYLCV. GO and KEGG pathway analysis implicated that these 15 whitefly proteins are of different biological functions/processes mainly including metabolic process, cell motility, signal transduction, and response to stimulus. We then found that the whitefly protein tumorous imaginal discs (Tid), one of the 15 whitefly proteins identified, had a stable interaction with TYLCV CP *in vitro*, and the DnaJ_C domain of Tid_301−499aa_ may be the viral binding site. During viral retention, the expression of whitefly protein Tid was observed to increase at the protein level, and feeding whiteflies with dsRNA or antibody against Tid resulted in a higher quantity of TYLCV in the whitefly body, suggesting the role of Tid in antiviral infection. Our data indicate that the induction of Tid following viral acquisition is likely a whitefly immune response to TYLCV infection.

## Introduction

Many plant viruses, such as species of the *Luteoviridae, Geminiviridae*, and *Nanoviridae* families, are transmitted by hemipteran insects in a persistent, circulative manner (1). During the long-term virus-vector interactions, insect vectors have developed two inevitable physical barriers to virus movement: midgut and salivary glands (1, 2). Initially, the vector ingests virions from virus-infected plants; then, virions enter the insect midgut lumen and subsequently cross through the midgut epithelial cells to be released into the hemolymph. Afterwards, virions move along with the hemolymph and reach the salivary glands from which they are injected into plants together with whitefly saliva secretion (1, 2). During this circulative journey, viruses need to interact with the insect vector in a coordinated manner for successful transmission to occur; at the same time, viral infection may activate immune reactions from its vector (3, 4).

Begomoviruses (genus *Begomovirus*, family *Geminiviridae*) are a group of single-stranded circular DNA viruses, which are transmitted by whiteflies of the *Bemisia tabaci* species complex in a circulative manner (5, 6). Some begomoviruses are serious viral disease agents of many crops worldwide. For example, tomato yellow leaf curl virus (TYLCV) is transmitted by a notorious invading species of whitefly, provisionally named as Middle East–Asia Minor 1 (MEAM1), of the *B. tabaci* species complex and has caused enormous damage to the production of tomato and some other crops in many countries/regions in the last three decades (7–9). Similar to other begomoviruses and other circulatively transmitted viruses, ingested TYLCV moves along the path of stylet-midgut-hemolymph-salivary glands in whitefly vectors. During the movement, TYLCV depends on clathrin-mediated endocytosis to enter the midgut cells and then accumulates in intracellular vesicle-like structures (10–12). At the same time, a viral infection activates the whitefly autophagy pathway, which plays an important role in the antiviral response (13, 14).

Up to now, the coat protein (CP) is the only structural protein of begomoviruses known to be involved in viral movement in the vector (15). CP gene replacement results in dramatic changes in characteristics of viral acquisition and transmission by whitefly vector (16–19). However, so far, only a few whitefly proteins have been reported to interact with the viral CP. The heat shock protein 70 (HSP70) and vesicle-associated membrane protein–associated protein B (VAPB) show inhibitory roles in virus transmission (20, 21), and a peptidoglycan recognition protein BtPGRP acts in whitefly immunity (22). In contrast, GroEL produced by secondary endosymbionts *Hamiltonella* or *Arsenophonus* may protect the virus from degradation in vector hemolymph (23, 24), and the midgut protein, cyclophilin B and collagen protein may assist in viral transmission (25–27). Vitellogenin may enable transovarial transmission of virus to the next generation of whitefly (28). The putative roles of *Bt*HSP16, thioredoxin-like protein (TLP) and protein BtR242 produced by *Rickettsia* in the viral transmission are yet unclear (29–31). Despite this progress, the functions of some of the abovementioned proteins require further validation, and many more vector components remain to be discovered to achieve an adequate understanding of begomovirus-whitefly interactions.

In this study, first, using the yeast two hybrid (Y2H) system, we identified 15 candidate whitefly proteins interacting with TYLCV CP, including the evolutionarily highly conserved protein tumorous imaginal discs (Tid). As the mammalian homolog of whitefly Tid has been implicated for its role in a variety of signaling pathways and autophagy (32, 33), we then conducted a series of molecular experiments and bioassays to examine *in vitro* interaction between whitefly Tid and TYLCV CP. Following viral infection, increase of whitefly Tid at the protein level exerted constraints on viral retention. Our data provide novel insights into begomovirus-whitefly interactions, indicating the negative impact of Tid on viral retention.

## Materials and Methods

### Virus, Plants, and Insects

TYLCV clone isolate SH2 (GenBank accession number: AM282874.1) was agro-inoculated into tomato plants (*Solanum lycopersicon* L. cv. Hezuo903) at the 3–4 true leaf stage. The tomato plants were then cultivated to the 7–8 true leaf stage, and plants showing typical symptoms were taken for use in experiments. Cotton plants (*Gossypium hirsutum* L. cv. Zhemian, 1793) were cultivated to the 6–7 true leaf stage for whitefly culture maintenance and experiments. A stock culture of MEAM1 whitefly was maintained in insect-proof cages on cotton plants at 26 ± 1°C, 60% relative humidity and 14 h light/10 h darkness.

### Y2H Assay System

The Y2H assay based on the matchmaker gold yeast two-hybrid system (Cat. No. 630489; Clontech) was used to explore the interactions between whitefly proteins and TYLCV CP. The cDNA library of whitefly was constructed in the prey plasmid of SfiI-digested pGADT7. The full-length of TYLCV CP gene was cloned into the bait plasmid of pGBKT7 after Nde I and EcoR I restriction. Primers used for cloning are listed in Supplementary Table 1. We used the following procedure for the Y2H assay: (1) transform the recombinant plasmid pGBKT7-TYLCV CP into the Y2H Gold yeast strain; (2) select the yeast strain on synthetic defined minimal medium lacking tryptophan (S.D./-Trp); (3) extract the yeast protein by yeast total protein extraction kit (Cat. No.C500013; Sangon Biotech) and confirm the expression of TYLCV CP in yeast in a Western blot by anti-TYLCV CP antibody (provided by Professor Jian-Xiang Wu); (4) conduct the auto-activation detection; (5) transform the cDNA library of whitefly into the Y2HGold yeast strain containing the bait plasmid pGBKT7-TYLCV CP; (6) observe the growth of yeast strain on the double dropout medium (DDO: S.D./-Leu/-Trp) and triple dropout medium (TDO: S.D./-His/-Leu/-Trp) with 40 μg/ml X-alpha-Gal and 125 ng/ml aureobasidin A (AbA) (TDO/X/A), select the positive clones on TDO/X/A; (7) restreak these positive clones on quadruple dropout medium (QDO: S.D./Ade/-His/-Leu/-Trp) with 40 μg/ml X-alpha-Gal and 125 ng/ml AbA (QDO/X/A) to eliminate the false positives; (8) recover the prey plasmids from the positive clones and transform them into *Escherichia coli* strain DH5α, sequence, and identify their interactions with TYLCV CP again. The different fragments screened from the whitefly cDNA library were used in a BLAST search of the NCBI database (http://blast.st-va.ncbi.nlm.nih.gov/Blast.cgi), and the sequences of these fragments screening in the Y2H assay were deposited in GenBank.

### Bioinformatic Analysis

Whitefly proteins identified from the Y2H assay system were categorized according to their gene ontology (GO) annotation using the Blast2GO software and then performed using the OmicShare tools, a free online platform for data analysis (http://www.omicshare.com/tools). The metabolic pathway analysis of these proteins was conducted according to the Kyoto Encyclopedia of Genes and Genomes (KEGG) pathway annotation (https://www.kegg.jp/blastkoala/). Network diagrams were created using the database search tool for the retrieval of interacting genes/proteins (STRING 9.1; http://stringdb.org). All of these analyses were conducted by the full length of amino acid sequences.

### Real-Time PCR

Quantitative (q) PCR was performed on CFX connect real-time PCR system (Bio-Rad, USA) using the SYBR Premix Ex Taq II (Cat. No. RR820A, Takara). β-Actin was used as an internal reference, and relative abundance of TYLCV or transcript was calculated by 2^−ΔΔCt^. Primers used for real-time PCR are listed in Supplementary Table 1.

### dsRNA Synthesis

DNA templates with a T7 promoter at both ends of selected genes were used to synthesize dsRNA following the manufacturer's instruction of the T7 high-yield transcription kit (Cat. No.TR101-02; Vazyme). Then, dsRNA was purified using phenol: chloroform extraction, isopropanol precipitation, and resuspended in nuclease-free water. The size and quality of the dsRNA were confirmed by 1% agarose gel electrophoresis, and its quantity was measured using Nanodrop (Thermo Scientific, USA). Ds*GFP* was used as a control. Primers used for DNA template synthesis are listed in Supplementary Table 1.

### Membrane Feeding on dsRNA or Antibody

Whitefly adults within 7 days post-emergence were collected from cotton plants. A group of 250 adults were released into a glass tube 1.5 cm in diameter and 10 cm in length. According to Pan et al. (10), for dsRNA silencing, whiteflies were fed on 15% sucrose solution containing 200 ng/μl dsRNA for 48 h, and 15% sucrose solution with the same amount ds*GFP* was used as control. For antibody feeding, Tid polyclonal antibody (PcAb) was mixed with 15% sucrose solution with a dilution rate of 1:50 for 24 h, and 15% sucrose solution with the same dilution of rabbit pre-immune serum was set as control.

### Viral Acquisition

For viral acquisition after ds*Tid* or ds*UBR7* feeding (ds*GFP* was used as control), whiteflies were caged with leaves from the same branch of TYLCV-infected tomato plants for 6, 12, or 24 h, respectively, and then transferred to feed on cotton for 48 h for viral retention. Female adults were collected in groups of 10 each and homogenized in 100 μL lysis buffer for relative viral quantity analysis (10). Three biological replicates were conducted for relative viral quantity analysis by real-time PCR. For the subsequent experiments of membrane feeding of dsRNA or antibody against Tid, whiteflies were caged with leaves of two symmetrical leaves of the same height on the same branch of TYLCV-infected tomato plants for 12 h and then transferred to feed on cotton for 48 h for viral retention. Three to five biological replicates were conducted for relative viral quantity analysis by real-time PCR.

### Structural and Phylogenetic Analysis of Protein Tid

The amino acid sequence of Tid fragment screened from the Y2H assay (Tid-S, GenBank: MT505751) was aligned with the Tid full-length (Tid-FL, GenBank: MT505750) using Clustal X (2.0). Phylogenetic reconstruction was conducted using the maximum likelihood (ML) method and the global transvers time (GTR) model implemented in the MEGA v.6 program (34). Support for the internal nodes of the trees was evaluated using the bootstrap method with 10,000 replicates. The protein domain, transmembrane region, and signal peptide predictions were conducted using the NCBI conserved domain database (CDD) (http://www.ncbi.nlm.nih.gov/Structure/cdd/wrpsb.cgi), TMHMM Server v. 2.0 (http://www.cbs.dtu.dk/services/TMHMM/) and SignalP 4.1 Server (http://www.cbs.dtu.dk/services/SignalP/), respectively. The 3-D structure of protein Tid was predicted using swissmodel (http://swissmodel.expasy.org).

### Full-Length Amplification, Protein Expression, and Antibody Production

The ORF of Tid-FL (GenBank: MT505750) was amplified from the whitefly cDNA using PrimerSTAR max DNA polymerase (Cat. No. R045A; Takara) and then cloned into pET28a plasmid for fusion with His tag. His-Tid-FL was expressed in inclusion bodies of *E. coli* strain *Rosetta*, and following renaturation and purification of inclusion body protein, His-Tid-FL was used to immunize rabbits to obtain a Tid-specific PcAb by HuaBio (China). Primers used in this experiment are listed in Supplementary Table 1. The specificity of Tid rabbit PcAb is shown in Supplementary Figure 1.

### Glutathione-S-Transferase (GST) Pull-Down

*Tid-S, Tid*_76−138*aa*_ (226-414 bp of *Tid-FL*, DnaJ domain), *Tid*_239−299*aa*_ [715-897 bp of *Tid-FL*, four repeats of a CXXCXGX(G) motif], and *Tid*_301−419*aa*_ (901-1,257 bp of *Tid-FL*, DnaJ_C domain) were cloned into pMAL-c5X for fusion with MBP tag, accordingly. TYLCV CP was cloned into pGEX-6p-1 for fusion with GST tag. These recombinant proteins were expressed in *E. coli* strain Rosetta and purified. GST-TYLCV CP was bound to glutathione agarose beads (Cat. No.17-5132-01; GE Healthcare) for 2–4 h at 4°C. Then the mixtures were centrifuged for 5 min at 1,000 rpm, and the supernatants were discarded. Agarose beads were washed five times with 1 × phosphate-buffer saline (PBS). Different purified and desalinated MBP-tag fusion proteins or the native whitefly proteins extracted by cytoplasmic extraction buffer (Cat. No.SC-003; Invent) were added to the beads, respectively, and incubated for 4 h at 4°C. These mixtures were centrifuged and washed five times with 1 × PBS, and the bead-bound proteins were eluted by boiling in PAGE buffer (Cat. No. FD 002; FDbio) for 10 min. Finally, these proteins were separated by SDS/PAGE gel electrophoresis and detected by Western blot using anti-MBP antibody (Cat. No. ab49923; Abcam) or anti-Tid antibody. Primers used are listed in Supplementary Table 1.

### Expression Analysis of Tid

Whitefly adults within 7 days post-emergence from cotton were transferred to TYLCV-infected tomato for 12 h and then transferred to feed on cotton for 48 h. Un-infected tomato was used as a control. Whitefly adults (three biological replicates) were collected as groups of 30 adults for analyzing gene expression of *Tid* at the transcriptional level. Total RNA of whitefly was isolated with TRIzol (Cat. No. 15596-026; Invitrogen), and reverse transcription was done using the PrimeScript RT reagent kit (Cat. No. DRR037A; Takara). For translational-level analysis, 100 whitefly adults were collected as one sample for protein extraction by RIPA (Cat. No. P0013B; Beyotime). Then, we used the BCA protein assay (Cat. No. 23250; Thermo Scientific) to determine and unify the concentration of protein samples. Western blot analysis was conducted by anti-Tid antibody, using anti-β-actin antibody (Cat. No. E021020-01; Earthox) as a control. The translational-level analysis was repeated three times, and ImageJ was used to quantify the relative protein level, Following ds*Tid* membrane feeding, 12 h viral acquisition, and 48 h viral retention, the expressions of Tid at transcriptional and translational levels were analyzed as described above.

### Statistical Analysis

Comparison of the relative abundance of virus in whitefly and expression levels of genes were performed using an independent *t*-test with *P* < 0.05 as the threshold of significant difference (^*^P < 0.05, ^**^P < 0.01, and ^***^P < 0.001). All the statistical analyses were performed using SPSS 20.0 (SPSS Inc., USA).

## Results

### Analysis of the Interactions Between Whitefly Proteins and TYLCV CP

**Figure 1:**
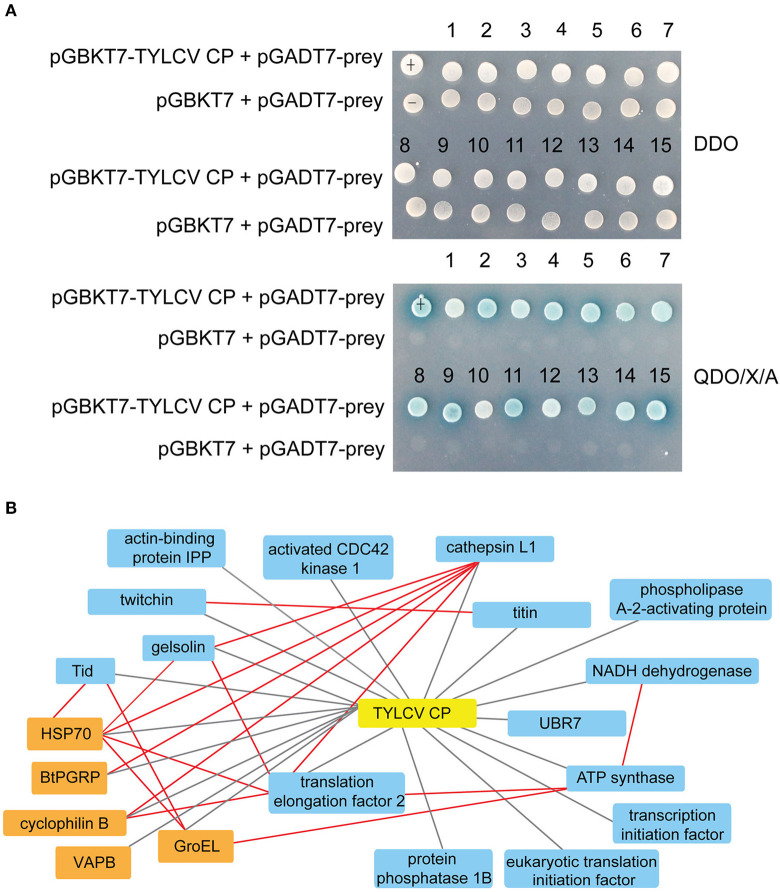
Interactions between whitefly proteins and TYLCV CP. **(A)** Confirmed interactions between TYLCV CP and screened proteins of *B. tabaci* using Y2H assay. TYLCV CP and 15 respective prey proteins were used to cotransform yeast for growth on DDO and QDO/X/A selective medium. pGBKT7-p53 and pGADT7-LargeT were used as positive controls (+); pGBKT7-p53 and pGADT7 were used as negative controls (–). **(B)** Protein interaction network was constructed using TYLCV CP and 20 whitefly protein homologs of *Drosophila melanogaster;* 20 whitefly proteins include 15 candidate whitefly proteins (blue) obtained in this study and five other whitefly proteins (orange) available in the literature related to MEAM1 whitefly-TYLCV interactions. The red line means the interaction predicted from the database search tool for the retrieval of interacting genes/proteins (STRING 9.1; http://stringdb.org), the black line stands for the interaction supported by experiments.

As shown in Supplementary Figure 2A, the Y2H system was used to examine the interactions between whitefly proteins and TYLCV CP. The titer of the primary whitefly cDNA library was ~5.0 × 10^6^ cfu with an average insert size of 1 kb, meeting the requirements of a standard cDNA library (Supplementary Figure 2B). The fusion expression of TYLCV CP with GAL4 DNA-BD in the yeast (≈46 kDa) was verified using Western blot analysis (Supplementary Figure 2C). The auto-activation detection showed that the bait plasmid pGBKT7-TYLCV CP could be used in this Y2H system (Supplementary Figure 2D). After Y2H screening, 26 positive clones were isolated, and 15 unique whitefly proteins were identified (Table 1). To identify the one-to-one interaction between bait and prey protein, the interactions between these 15 screened whitefly proteins and TYLCV CP were validated using the Y2H assay (Figure 1A), combined with reported interactions between MEAM1 whitefly and TYLCV, and a protein interaction network was generated, including the predicted interactions among whitefly proteins (Figure 1B).

**Table 1:** Putative interacting proteins with TYLCV CP in whitefly by the Y2H screen.

**No**.	**GenBank accession^a^**	**NCBI reference sequence^b^**	**Identity (%)^c^**	**Protein name**
1	MT505752	XP_018911927.1	94	ATP synthase subunit beta, mitochondrial
2	MT505751	XP_018917603.1	54	Protein tumorous imaginal discs, mitochondrial-like
3	MT505753	XP_018912446.1	59	Gelsolin-like isoform X2
4	MT505754	XP_018903232.1	49	Actin-binding protein IPP-like
5	MT505755	XP_018902418.1	53	Eukaryotic translation initiation factor 4H isoform X2
6	MT505756	XP_018896959.1	65	Titin isoform X14
7	MT505757	XP_018903674.1	59	Twitchin isoform X10
8	MT505758	XP_018899978.1	59	Transcription initiation factor TFIID subunit 1-like
9	MT505759	XP_018904341.1	88	Translation elongation factor 2
10	MT505760	XP_018900124.1	48	NADH dehydrogenase [ubiquinone] 1 beta subcomplex subunit 9
11	MT505761	XP_018914181.1	61	Protein phosphatase 1B
12	MT505762	XP_018910197.1	35	Phospholipase A-2-activating protein
13	MT505763	XP_018905030.1	44	Putative E3 ubiquitin-protein ligase UBR7
14	MT505764	XP_018911353.1	65	Cathepsin L1
15	MT505765	XP_018904178.1	36	Activated CDC42 kinase 1

a *Sequences of whitefly genes obtained in this study, they were partial CDS which were deposited in GenBank*.

b NCBI reference full-length sequences of whitefly genes screening in Y2H assay. The full length of protein Tid was also obtained in this work (GenBank: MT505750), sharing 100% amino acid identity with its NCBI reference sequence (XP_018917603.1).

c *Identity: amino acid identity of whitefly proteins with Drosophila melanogaster counterpart*.

### *In silico* Analysis of the Whitefly Proteins Screened by Y2H Assay

**Figure 2:**
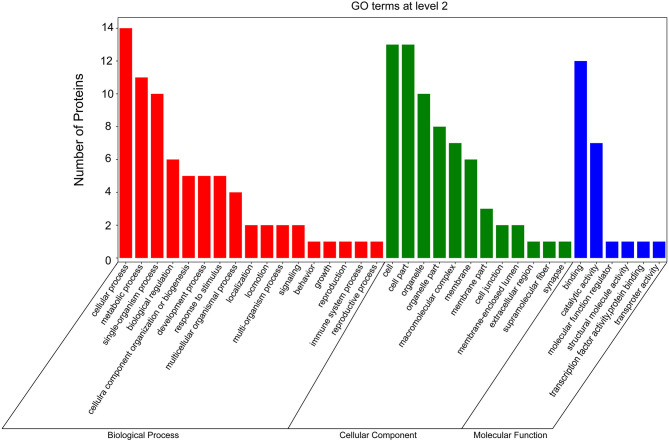
GO analysis of the 15 putative interactors inferred via screening in the Y2H system. Different colors represent different GO categories. GO annotation was conducted by the Blast2GO software, and the figure was generated using the OmicShare tools, a free online platform for data analysis (http://www.omicshare.com/tools).

**Figure 3:**
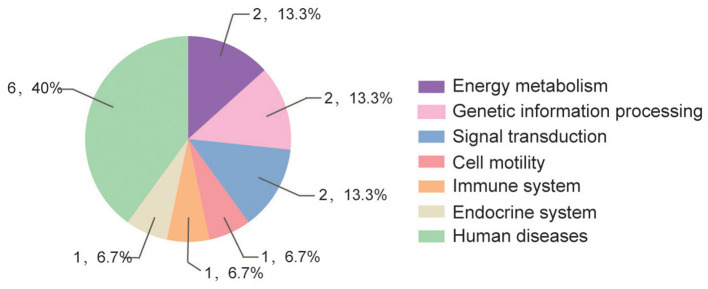
Pathway distribution of the 15 putative interactors inferred via screening in the Y2H system. The metabolic pathway analysis of these proteins was conducted according to the Kyoto Encyclopedia of Genes and Genomes (KEGG) pathway annotation (https://www.kegg.jp/blastkoala/); in total, 15 pathways were identified for the 15 prey proteins.

According to GO and KEGG analyses (Figures 2, 3), the 15 interactors from the Y2H assay (Table 1) were classified into different groups, mainly including metabolic process, cell motility, signal transduction, and response to stimulus. The GO analysis suggests that the 15 proteins may be responsible for 17 different biological processes, mainly involved in cellular and metabolic processes with different distributions inside and outside of cells; most of them shared the binding activity, and about half of them possess catalytic activity (Figure 2). The KEGG pathway analysis suggests that the 15 proteins can be classified into 7 groups (Figure 3). For example, gelsolin-like isoform X2 belongs to the pathway of cell motility; protein phosphatase 1B as a member of the MAPK signaling pathway belongs to the group of signal transduction. The whitefly autophagy pathway and ubiquitin-proteasome system have been shown to play a role in antiviral response (13, 14, 35). Among the 15 whitefly proteins, there is a ubiquitin-protein ligase (UBR7) and a protein Tid related to macro-autophagy (33). Both of these two proteins belong to the biological process of response to stimulus (GO: 0050896).

### Effects of dsRNA Interference of Tid and UBR7 on Viral Retention

**Figure 4:**
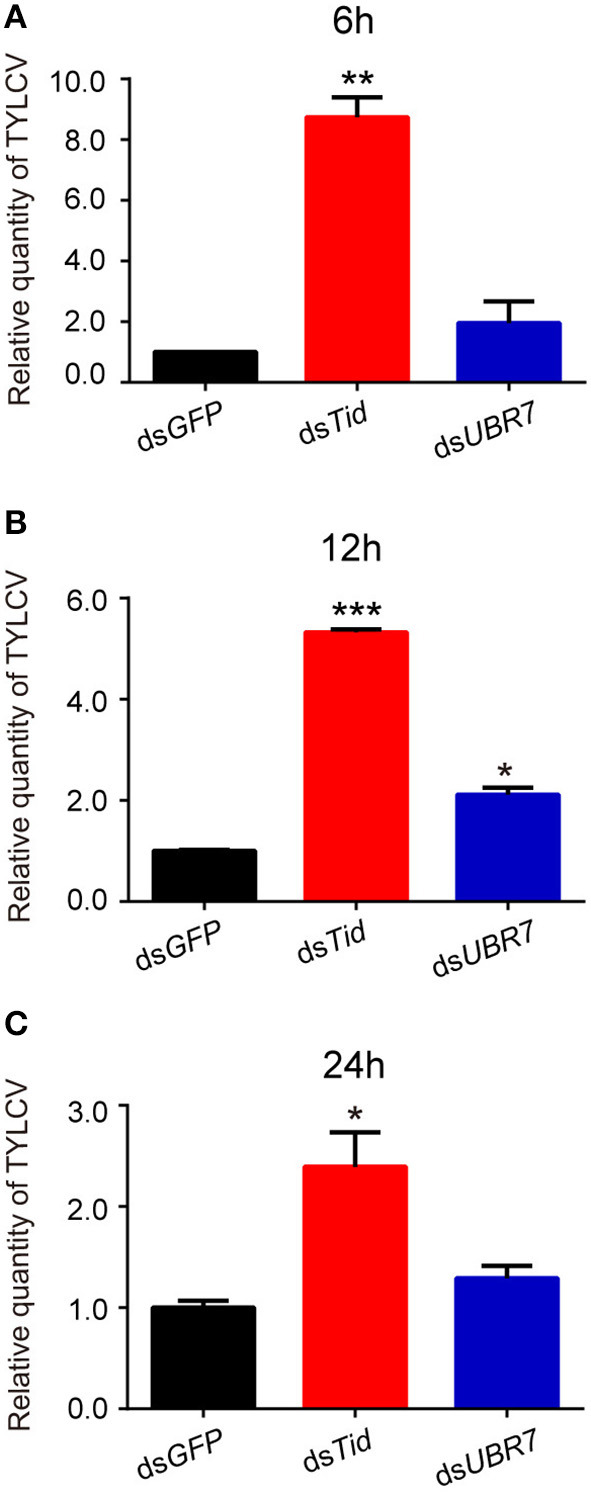
Effects of dsRNA interference of *Tid* and *UBR7* on TYLCV retention. After dsRNA feeding, whiteflies of the interference treatments were caged to feed on leaves of the same branch of a TYLCV-infected tomato plant for 6 h **(A)**, 12 h **(B)**, or 24 h **(C)**, and then transferred to feed on cotton for 48 h to test the viral retention ability of the whitefly by qPCR. *GFP* was used as a control. Whitefly females were collected in groups of 10 each and homogenized in 100 μL lysis buffer for relative viral quantity analysis. In **(A)**, ds*Tid*: *n* = 3, *t* = −11.716, *P* = 0.0072; ds*UBR7*: *n* = 3, *t* = −1.327, *P* = 0.3158; in **(B)**, ds*Tid*: *n* = 3, *t* = −66.018, *P* < 0.0001; ds*UBR7*: *n* = 3, *t* = −7.855, *P* = 0.0138; and in **(C)**, ds*Tid*: *n* = 3, *t* = −4.035, *P* = 0.0157; ds*UBR7*: *n* = 3, *t* = −2.058, *P* = 0.1087. Independent *t*-test was used here and the differences between treatments were considered significant when *P < 0.05; **P < 0.01, ***P < 0.001.

To examine the role of proteins Tid and UBR7 on virus retention, whiteflies that had received dsRNA interference treatment were transferred to feed on a TYLCV-infected tomato for 6, 12, or 24 h, respectively, and then transferred to feed on cotton for 48 h. At the end of each of the three time points, after ds*Tid* interference, the relative viral quantity in whiteflies significantly increased compared to the control (Figures 4A–C) although, for UBR7 dsRNA interference, following a viral acquisition access period for 12 h, the defense ability of the whitefly against TYLCV retention significantly decreased (Figure 4B). When the intervals of the viral acquisition access period lasted 6 or 24 h, the defense ability of the whitefly against TYLCV retention had non-significant decrease (Figures 4A,C). Based on these results, we selected Tid for the following experiments.

### Structural and Phylogenetic Analysis of the Protein Tid

**Figure 5:**
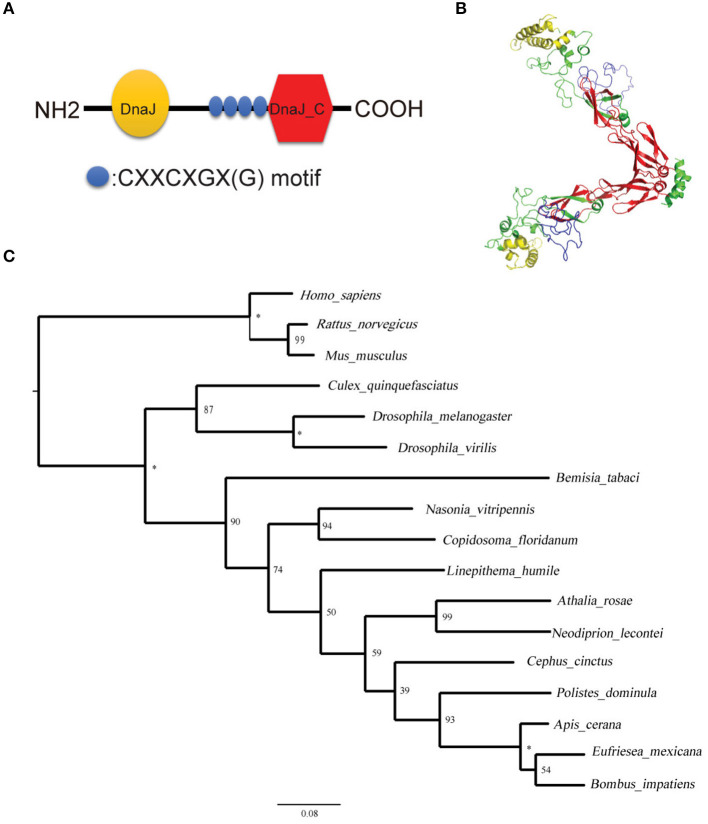
Structural and phylogenetic analysis of the protein Tid. **(A)** Graphic presentation of the Tid structure. **(B)** 3-D structure of protein Tid. Asterisk indicates that the nodes were 100% supported. **(C)** Phylogenetic tree of *B. tabaci*-Tid and other arthropods and mammals were constructed using MEGA v.6 with the maximum likelihood (ML) method. Numbers next to the branches indicated bootstrap value of each internal branch in the phylogenetic tree nodes from 10000 replicates. Tid sequences include *Bemisia tabac*i (MT505750), *Homo sapiens* (NM 001286516), *Rattus norvegicus* (NM 001038596), *Mus musculus* (NM 001135112), *Culex quinquefasciatus* (XM 001848856), *Drosophila melanogaster* (NM 001259554), *Drosophila virilis* (XM 002059276), *Bemisia tabaci* (XP 018917603), *Nasonia vitripennis* (XM 016983982), *Copidosoma floridanum* (XM 014350678), *Linepithema humile* (XM 012379048), *Athalia rosae* (XM 012406140), *Neodiprion lecontei* (XM 015659368), *Cephus cinctus* (XM 015737810), *Polistes dominula* (XM 015322978), *Apis cerana* (XM 017059752), *Eufriesea mexicana* (XM 017897896), and *Bombus impatiens* (XM 012384798).

After sequencing the Tid prey plasmid screened from Y2H, we obtained an 855 bp long (285 aa) Tid-S sequence (GenBank: MT505751), having a 60% coverage (164–448aa) of Tid-FL. Tid-FL (GenBank: MT505750, ≈52 kDa) has a DnaJ domain (N-terminal, 76–138aa), a DnaJ_C domain (C-terminal, 301–419aa), and four repeats of a CXXCXGX(G) motif (239–299aa). Tid-S contains only the CXXCXGX(G) motifs and DnaJ_C domain (Figure 5A). Tid-FL has no transmembrane domain or signal peptide, and its 3-D structure model is shown in Figure 5B. Phylogenetic analysis of *B. tabaci* Tid and 16 other insect Tid proteins showed that *B. tabaci* Tid forms a monophyletic lineage with species of Hymenoptera and appears closely related to the genus *Drosophila* (Figure 5C). This DnaJ domain-containing protein is evolutionarily highly conserved; Tid in mammals and that of *Drosophila* show 54.9% identity in amino acid sequences (36), and the Tid of whitefly and that of *Drosophila melanogaster* show 54.0% identity in amino acid sequences.

### *In vitro* Evidence Supports the Interaction Between Tid and TYLCV CP

**Figure 6:**
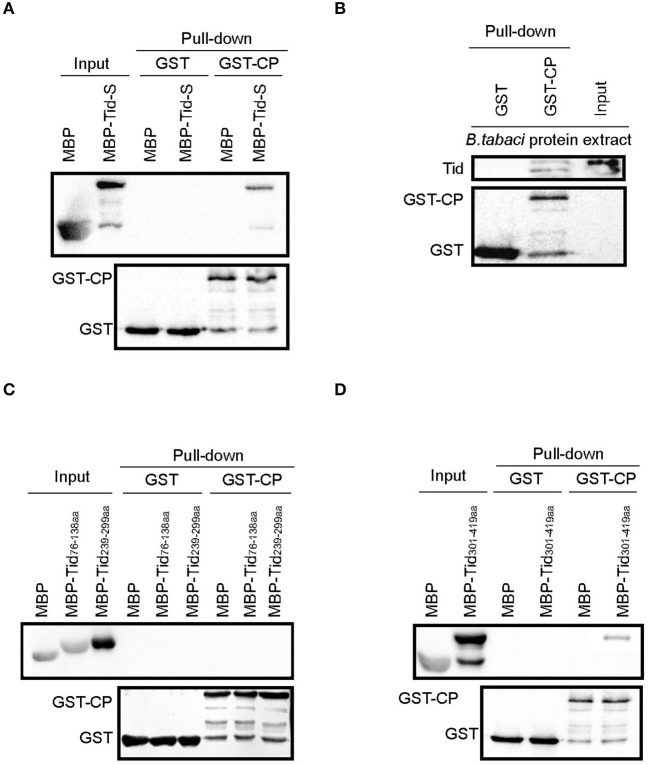
Interaction analysis between Tid and TYLCV CP. GST-TYLCV CP was used as bait protein, **(A)** MBP-Tid-S was used as prey protein, confirmation of the interaction between GST-TYLCV CP and MBP-Tid-S using GST pull-down; **(B)** native whitefly proteins extracted by cytoplasmic extraction buffer (Cat. No.SC-003; Invent) were used as prey proteins, identification of the interaction between whitefly endogenous Tid and GST-TYLCV CP by GST pull-down; **(C)** MBP-Tid_76−138aa_ and MBP-Tid_239−299aa_ were, respectively, used as prey protein, the interactions between these two Tid regions and GST-TYLCV CP were conducted via GST pull-down. **(D)** The C terminal of Tid_301−419aa_ was used as prey protein, identification of the interaction between Tid_301−419aa_ and GST-TYLCV CP via GST pull-down.

TYLCV CP fused with GST and Tid-S tagged with MBP were used to verify their interaction through GST pull-down analysis (Figure 6A). Using the fusion protein GST-TYLCV CP as a bait protein and native whitefly proteins extracted by cytoplasmic extraction buffer (Cat. No.SC-003; Invent) as prey proteins, whitefly endogenous Tid could co-elute with GST-fused TYLCV CP but not with GST (Figure 6B). Further, we tested the interaction between TYLCV CP and different regions of Tid-FL mentioned above: Tid_76−138aa_ (DnaJ domain), Tid_239−299aa_ (four repeats of a CXXCXGX(G) motif), and Tid_301−419aa_ (DnaJ_C domain). The results showed that Tid_76−138aa_ and Tid_239−299aa_ show no binding activity with TYLCV CP (Figure 6C); the binding site of TYLCV CP may be located in the C terminal of Tid-FL (Figure 6D).

### The Increase of Tid at Protein Level During Viral Retention

**Figure 7:**
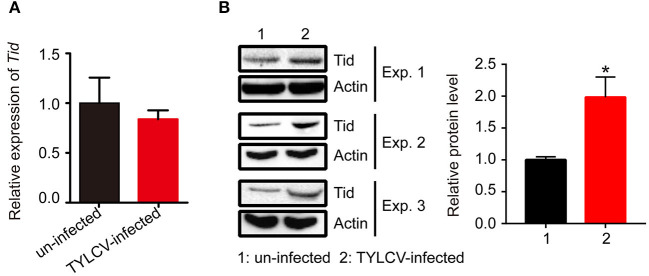
The expression of Tid following TYLCV acquisition. **(A)** The change of the *Tid* expression following viral acquisition at transcriptional level was analyzed by qPCR analysis (*n* = 3, *t* = 0.594, *P* = 0.5842). **(B)** The expression of Tid following viral acquisition at protein level was analyzed by Western blot analysis; 100 whitefly adults were collected as one sample for protein extraction and BCA protein assay was used to determine and unify the concentration of protein samples. Three biological replicates were set, and the results were quantified by ImageJ, *t* = −3.077, *P* = 0.0370. Independent *t*-test was used here and the differences between treatments were considered significant when *P < 0.05.

Following viral infection, the expression of Tid at both transcriptional and translational levels was tested. Data demonstrates that there was no significant change of the expression of *Tid* at transcriptional level (Figure 7A). However, Western blot analysis showed TYLCV infection could significantly increase the expression of Tid at protein level (Figure 7B).

### Effects of Tid Interference on TYLCV Retention

**Figure 8:**
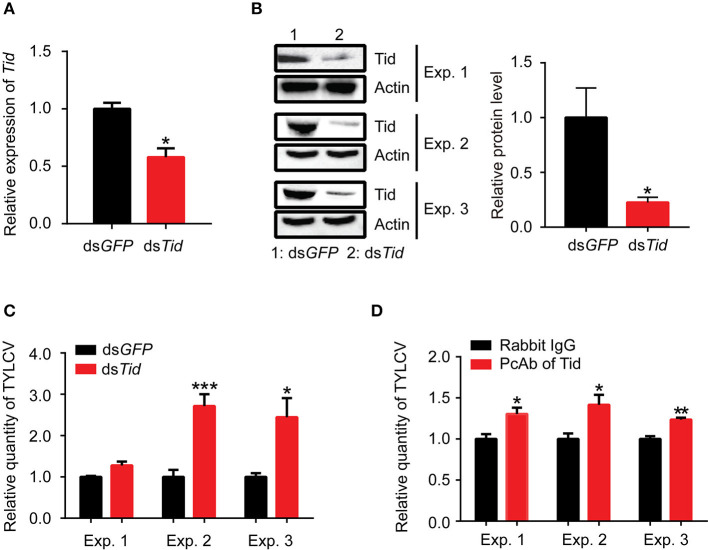
Tid restricts viral retention in whiteflies. After feeding with dsRNA, **(A)** *Tid* mRNA levels after were analyzed by qPCR analysis. Whitefly adults were collected as groups of 30 adults for RNA isolation and cDNA synthesis (*n* = 3, *t* = 4.46, *P* = 0.0111); **(B)** Tid protein levels were analyzed by Western blot analysis; 100 whitefly adults were collected as one sample for protein extraction and BCA protein assay was used to determine and unify the concentration of protein samples. Three biological replicates were set and the results were quantified by ImageJ, *t* = 2.82, *P* = 0.0478. **(C)** After ds*Tid* interference and viral acquisition, TYLCV levels in whitefly whole body was analyzed by qPCR analysis. Whitefly females were collected in groups of 10 each and homogenized in 100 μL lysis buffer for relative viral quantity analysis (Exp. 1, *n* = 4–5, *t* = −2.85, *P* = 0.0565; Exp. 2, *n* = 5, *t* = −5.12, *P* = 0.0009; Exp. 3, *n* = 3, *t* = −3.06, *P* = 0.0375). **(D)** Effect of feeding whiteflies with an antibody against Tid: quantity of virus in the whole body (Exp. 1, *n* = 5, *t* = −3.11, *P* = 0.0145; Exp. 2, *n* = 4, *t* = −2.96, *P* = 0.0252; Exp. 3, *n* = 3–4, *t* = −5.32, *P* = 0.0031). Independent *t*-test was used here and the differences between treatments were considered significant when *P < 0.05; **P < 0.01, ***P < 0.001.

Following dsRNA feeding, the adults were transferred to feed on TYLCV-infected tomato plants for 12 h for virus acquisition and then were transferred to feed on cotton for 48 h for observation on virus retention. Data showed that the expression of *Tid* in whiteflies was effectively knocked down via dsRNA interference (Figures 8A,B), and knockdown of *Tid* expression resulted in significant increases of relative virus quantity in whiteflies (Figure 8C). In addition, blocking Tid function by anti-Tid antibody likewise resulted in significantly higher relative virus quantity in whiteflies during virus retention (Figure 8D).

## Discussion

Investigation of the interactions between begomoviruses and whitefly proteins can provide new knowledge of the virus transportation journey in vector. In this study, 15 candidate whitefly proteins of various categories were detected that may interact with TYLCV CP. In further tests of Tid and UBR7, two of the 15 candidate proteins detected showed that both proteins posed an adverse effect on viral retention, and Tid had a stronger effect than UBR7. A stable interaction between whitefly Tid and TYLCV CP was then observed, and the C-terminal of Tid was observed to be the likely binding site. Viral infection could increase the expression of whitefly Tid at the protein level; feeding whiteflies with dsRNA or antibody against Tid resulted in a significantly higher quantity of TYLCV in the body of whiteflies following viral acquisition. Altogether, these data reveal one novel whitefly protein that may function in antiviral response.

The insect innate immune system incurs physical, cellular, and humoral responses to invaders (37), and it is common for insect vectors to take advantage of their immunity to fight against viral infection. Wang et al. (22) demonstrate that whitefly protein *Bt*PGRP with antibacterial activity acts in multiple immune-response functions. Wang et al. (38) show that insect vectors could operate the c-Jun N-terminal kinase (JNK) signaling pathway for controlling viral transmission, causing a significant reduction in virus accumulation and transmission. The studies of Luan et al. (13) and Wang et al. (14) indicate that autophagy is involved in whitefly repression of begomovirus infection and triggers complex interactions between virus and insect vector. A previous study reported a mammalian homolog of whitefly Tid, which acted as a key regulator in mediating autophagy independently of HSP70 (33). Data available to date indicate that both Tid and HSP 70 play a role in repressing virus infection [(20); this study]; however, the relationships among whitefly autophagy, Tid, HSP70, and TYLCV CP remain unclear. Molecular mechanisms underlying the activation of autophagy pathway by TYLCV-infection in whiteflies warrant further investigation. Our findings provide clues for future studies on these issues.

Additionally, the roles of other candidate proteins detected in this study are also worth exploring. Gelsolin is a key regulator of actin filament assembly and disassembly (39), and actin has been shown to interact with several viral proteins and plays important roles in viral transmission. For example, interactions between non-structural protein Pns10 of rice dwarf virus and the cytoplasmic actin of leafhoppers is correlated with insect vector specificity (40); the non-structural protein P7-1 of reovirus southern rice black-streaked dwarf virus generates tubules and this tubules associate with the actin cytoskeleton in insect vector (*Sogatella furcifera*) cells (41, 42). In addition, MAPK signaling pathway is known to be activated by a diverse group of viruses and has important roles in viral replication (43), such as supporting assembly and maturation of West Nile virus and dengue virus (44, 45), regulating multiple steps of influenza A virus replication (46) and so on. In view of the potential role of protein phosphatase in regulating the life cycle of Simian Virus 40 (47), a study of the relationship of protein phosphatase 1B (a member of the MAPK signaling pathway) with TYLCV infection may be worthwhile. These investigations may lead to a comprehensive recognition of whitefly binding partners of viral CP and better understanding of the complex interactions between begomoviruses and their whitefly vectors.

## Data Availability Statement

All datasets generated for this study are included in the article/Supplementary Material.

## Author Contributions

JZ and TG designed this study and conducted most experiments as well as data analysis. JZ drafted and revised the manuscript. TL did the qPCR analysis and participated in statistical analysis. J-CZ made bioinformatic analysis. FW participated in Y2H screening. X-WW participated in manuscript preparation. S-SL provided supervision for the study and participated in manuscript preparation and revision. All authors read and approved the final version of the manuscript.

## Conflict of Interest

The authors declare that the research was conducted in the absence of any commercial or financial relationships that could be construed as a potential conflict of interest. The reviewer JN-C declared a past co-authorship with several of the authors SS-L and XW-W to the handling editor.
