# Supplementary material for: Proteomic Analyses of Whitefly-Begomovirus Interactions Reveal the Inhibitory Role of Tumorous Imaginal Discs in Viral Retention
Source: Front Immunol. 2020 Aug 4;11:1596. doi: 10.3389/fimmu.2020.01596 (PMC7417349; doi:10.3389/fimmu.2020.01596)
Supplement: Supplementary file 1 [file Data_Sheet_1.docx]

Supplementary Material


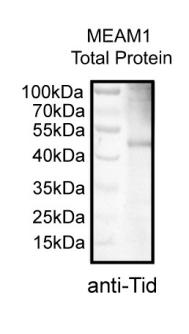


**Supplementary Figure 1.** The specificity of anti-Tid antibody. *B. tabaci* ptoteins were extracted by RIPA (Cat. No. P0013B; Beyotime) and then detected by western blot using Tid rabbit PcAb. The molecular weight of whitefly Tid is about 52 kda.


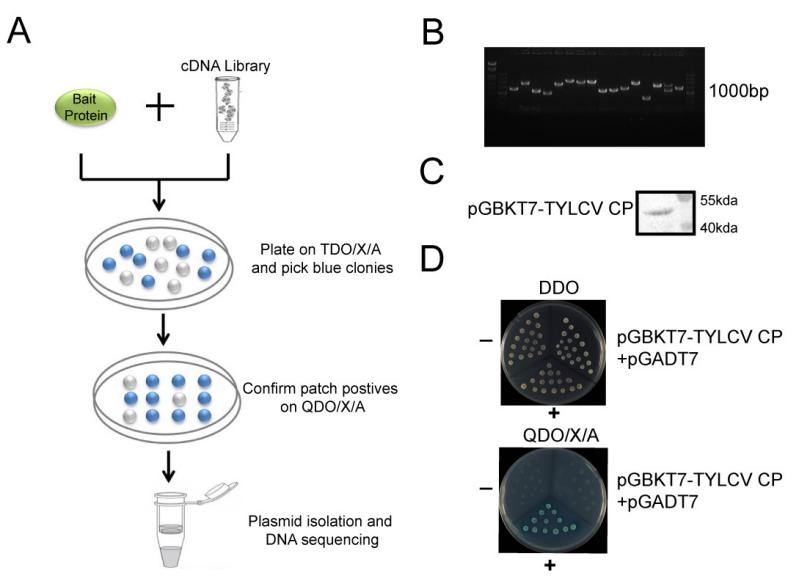


**Supplementary Figure 2**. Detecting proteins of *B. tabaci* that interact with TYLCV CP using the Y2H system. (A) Schematic diagram of workflow for the system. (B) PCR identification of inserts in the primary cDNA library for *B. tabaci*. (C) Expression of pGBKT7-TYLCV CP in yeast was identified using western blot by antibody of TYLCV CP (≈ 46 kda). (D) Auto-activation detection of bait vector pGBKT7-TYLCV CP in yeast. Gene activation as shown by growth on selective medium. When pGBKT7-TYLCV CP and pGADT7 were co-expressed, yeast transformants did not grow on the QDO/X/A selective medium. pGBKT7-p53 and pGADT7-LargeT were used as positive controls; pGBKT7- p53 and pGADT7 were used as negative controls.

**Supplementary Table 1.** Primers used in this study.

| Primer Name | Sequence |
| --- | --- |
| TYLCV CP-pGBKT7-F | GGGAATTCCATATGATGTCGAAGC |
| TYLCV CP-pGBKT7-R | CCGGAATTCTTAATTTGATAT |
| Tid-FL-pET28a-F | ATAAGAATGCGGCCGCATGGCAGTAACTCGG |
| Tid-FL-pET28a-R | GGGAATTCCATATGTTAGCCGAAAAGA |
| Tid-S-pMAL-C5x-F | GGGAATTCCATATGATGGGATCCTACCAAACT |
| Tid-S-pMAL-C5x-R | ATAGTTTAGCGGCCGCTTATTTGGTGGCATCAA |
| Tid_76-138aa_-pMAL-C5x-F | GGAATTCCATATGATGGATTACTATGATATCCT |
| Tid_76-138aa_-pMAL-C5x-R | CGCGGATCCTTAGTCATATTGCTTCCTTTTTGT |
| Tid_239-299aa_-pMAL-C5x-F | CGCGGATCCATGTGTCCCAGGTGTAAAGG |
| Tid_239-299aa_-pMAL-C5x-R | CCGGAATTCTTAGCCTTTGCCCGCACATT |
| Tid_301-419aa_-pMAL-C5x-F | CGCGGATCCATGAAAGTTGTAGCAAAAAGGA |
| Tid_301-419aa_-pMAL-C5x-R | CCGGAATTCTTATAAAGACCCTGGTGCTTTG |
| TYLCV CP-pGEX-6p-1-F | CGCGGATCCATGTCGAAGCGACCAGG |
| TYLCV CP-pGEX-6p-1-R | CCGGAATTCTTAATTTGATATTGAATCATA |
| TYLCV-qPCR-F | GAAGCGACCAGGCGATATAA |
| TYLCV-qPCR-R | GGAACATCAGGGCTTCGATA |
| Tid-qPCR-F | CCTGGGACCTCTTCACATCA |
| Tid-qPCR-R | ATCTTCTTCCAGCTCTGCGT |
| β-Actin-qPCR-F | TCTTCCAGCCATCCTTCTTG |
| β Actin-qPCR-R | CGGTGATTTCCTTCTGCATT |
| GFP T7-F | TAATACGACTCACTATAGGGAGACCACTGACCCTGAAGTTCATCTGC |
| GFP T7-R | TAATACGACTCACTATAGGGAGACCACGTCTTGTAGTTGCCGTCGTC |
| Tid T7-F | TAATACGACTCACTATAGGGAGACCACTCCCTCCTGGGGAAGGATTA |
| Tid T7-R | TAATACGACTCACTATAGGGAGACCACACAATCCTTTTTGCTACAAC |
| STAT5B T7-F | TAATACGACTCACTATAGGGAGAGTCGTTCCAGAAAAGGTTCC |
| STAT5B T7-R | TAATACGACTCACTATAGGGAGAGTAAATGGTTGTAACATGAC |
| UBR T7-F | TAATACGACTCACTATAGGGAGAGTGGAACTCAGGTATTCAATGG |
| UBR T7-R | TAATACGACTCACTATAGGGAGAGGGAGGAAGATTCTCAGGATT |
